# Large Language Models in Gastroenterology: Systematic Review

**DOI:** 10.2196/66648

**Published:** 2024-12-20

**Authors:** Eun Jeong Gong, Chang Seok Bang, Jae Jun Lee, Jonghyung Park, Eunsil Kim, Subeen Kim, Minjae Kimm, Seoung-Ho Choi

**Affiliations:** 1 Department of Internal Medicine, Hallym University College of Medicine Chuncheon Republic of Korea; 2 Institute for Liver and Digestive Diseases Hallym University Chuncheon Republic of Korea; 3 Institute of New Frontier Research Hallym University College of Medicine Chuncheon Republic of Korea; 4 Meninblox, Inc Gwangju Republic of Korea; 5 Tech University of Korea Siheung Republic of Korea; 6 Hansung University Seoul Republic of Korea

**Keywords:** large language model, LLM, deep learning, artificial intelligence, AI, endoscopy, gastroenterology, clinical practice, systematic review, diagnostic, accuracy, patient engagement, emotional support, data privacy, diagnosis, clinical reasoning

## Abstract

**Background:**

As health care continues to evolve with technological advancements, the integration of artificial intelligence into clinical practices has shown promising potential to enhance patient care and operational efficiency. Among the forefront of these innovations are large language models (LLMs), a subset of artificial intelligence designed to understand, generate, and interact with human language at an unprecedented scale.

**Objective:**

This systematic review describes the role of LLMs in improving diagnostic accuracy, automating documentation, and advancing specialist education and patient engagement within the field of gastroenterology and gastrointestinal endoscopy.

**Methods:**

Core databases including MEDLINE through PubMed, Embase, and Cochrane Central registry were searched using keywords related to LLMs (from inception to April 2024). Studies were included if they satisfied the following criteria: (1) any type of studies that investigated the potential role of LLMs in the field of gastrointestinal endoscopy or gastroenterology, (2) studies published in English, and (3) studies in full-text format. The exclusion criteria were as follows: (1) studies that did not report the potential role of LLMs in the field of gastrointestinal endoscopy or gastroenterology, (2) case reports and review papers, (3) ineligible research objects (eg, animals or basic research), and (4) insufficient data regarding the potential role of LLMs. Risk of Bias in Non-Randomized Studies—of Interventions was used to evaluate the quality of the identified studies.

**Results:**

Overall, 21 studies on the potential role of LLMs in gastrointestinal disorders were included in the systematic review, and narrative synthesis was done because of heterogeneity in the specified aims and methodology in each included study. The overall risk of bias was low in 5 studies and moderate in 16 studies. The ability of LLMs to spread general medical information, offer advice for consultations, generate procedure reports automatically, or draw conclusions about the presumptive diagnosis of complex medical illnesses was demonstrated by the systematic review. Despite promising benefits, such as increased efficiency and improved patient outcomes, challenges related to data privacy, accuracy, and interdisciplinary collaboration remain.

**Conclusions:**

We highlight the importance of navigating these challenges to fully leverage LLMs in transforming gastrointestinal endoscopy practices.

**Trial Registration:**

PROSPERO 581772; https://www.crd.york.ac.uk/prospero/

## Introduction

### Background

In the rapidly evolving landscape of health care, the convergence of medicine and technology has opened new avenues for improving patient care and operational efficiency. Among the most promising technological advancements is the development of artificial intelligence (AI), particularly large language models (LLMs), which have the potential to transform various medical specialties. The advent of AI has ushered in a new era of innovation across various sectors, with health care being a primary beneficiary [1-3].

Gastrointestinal endoscopy, a critical field for diagnosing and treating digestive diseases, stands on the cusp of significant advancements with the integration of these technologies. This, pivotal in diagnosing and treating digestive tract diseases, faces challenges like diagnostic variability and labor-intensive documentation. Gastrointestinal endoscopy, traditionally reliant on the expertise of specialists to interpret complex visual data and execute precise interventions, can greatly benefit from the automation and analytical capabilities provided by LLMs [4,5]. While traditional convolutional neural network (CNN)–based lesion detection or lesion diagnosis models in endoscopy have addressed the limitations of endoscopists’ visual diagnosis, LLMs capable of processing massive datasets are expected to address a wider range of clinical unmet needs by enhancing diagnostic support, automating report generation, and improving educational tools [1-3]. The AI models built thus far are narrow AI models designed to tackle a specific task for a specific purpose, and they have demonstrated very high performance within the scope of the problem they are intended to solve. Nevertheless, performance drops when the task’s objective or the type of data changes, necessitating a repeat of the data collection and model-improvement procedure in order to address the issue. Rapid advances in generative AI and the emergence of foundation models have led to the possibility of performing a wide range of tasks with no or minimal additional training.

### Brief History of LLMs

The inception of LLMs traces back to the early developments in AI and natural language processing. Initially, these models were designed to understand and generate human-like text by learning from vast datasets of text on the internet. The breakthrough came with the introduction of the Transformer architecture in 2017, which enabled models to handle long-range dependencies in text, significantly improving their understanding and generation capabilities. This led to the development of models like GPT and Bidirectional Encoder Representations from Transformers (BERT), which demonstrated unprecedented performance in a wide range of natural language processing tasks. LLM has been trained with tens to hundreds of billions of parameters using very large amounts of data, and it is mainly trained based on a pretext task that predicts the next word or token using a list of given words or tokens as input. One of the most defining characteristics of the LLM is the emergent ability of the model to grow in size, even when it is not trained for a specific task [6]. It has the ability to perform zero-shot learning, where it performs a task without examples, as well as few-shot learning, where its performance increases when a few examples are provided. These advancements paved the way for the integration of LLMs into various sectors, including health care, where they have been instrumental in enhancing diagnostic accuracy, automating documentation, and facilitating patient care. The evolution of LLMs has been marked by continuous improvements in model architecture, training techniques, and dataset quality, culminating in the current generation of models that are capable of complex reasoning and generating coherent, contextually relevant text.

### Prompt Engineering and Fine-Tuning

LLMs are pretrained models, and the learning process can be broadly divided into 2 parts: pretraining and fine-tuning. In the pretraining phase, the model is trained using large amounts of unstructured textual data (eg, news articles and novels), while in the fine-tuning phase, the pretrained model is fine-tuned for a specific task (model updates). In this fine-tuning phase, a small amount of labeled data is used to tune the model. In this step, the parameters of the model are fine-tuned to achieve optimal results for a specific task.

In-context learning is achieved through a technique called “prompt engineering” (no model updates). When a user enters a prompt, an LLM analyzes the text to understand the context and generate relevant output, that is, it performs the task you want it to do based on the content of the prompt alone. Literally, it means that the model understands (learns) the contextual meaning of the prompt (in-context) and generates an answer to it [6]. In-context learning does not update the weight of the model like pretraining or fine-tuning, and there is no separate model training process. Therefore, the importance of prompt engineering is emphasized because well-written prompts lead to good results. Depending on the number of examples, it can be divided into zero-shot, one-shot, and few-shot learning. One hypothesis for this unsupervised learning outcome is that the process of “inference” can be a form of “optimization” (maximum likelihood estimation) [7]. This systematic review aims to explore the emerging role of LLMs in gastroenterology, especially for gastrointestinal endoscopy, offering insights into how they can support specialists in improving diagnostic accuracy, streamlining documentation, enhancing training, and engaging patients.

## Methods

### Study Design

A systematic review was performed to check the recent research trend of the potential role of LLMs in gastrointestinal endoscopy. The protocol of this systematic review was registered at PROSPERO (581772) before the initiation of this study. The PICO (population, intervention, comparator, and outcome) was as follows: population: general population or patients with gastrointestinal disorders; intervention: application of LLMs; comparator: none; and outcome: clinical benefits or improvement of performance. This systematic review was performed in accordance with the statement of the PRISMA (Preferred Reporting Items for a Systematic Review and Meta-Analyses) for systematic review [8] (Multimedia Appendix 1).

### Databases

MEDLINE (through PubMed), Embase, and CENTRAL in the Cochrane Library were searched using common keywords (from inception to April 2024). Medical Subject Headings terminology or Emtree keywords were used for the search strategy. Two evaluators (EJG and CSB) independently performed literature searching, and disagreements between the 2 evaluators were resolved by discussion or consultation with a third author (JJL).

In detail, duplicated papers were first removed from the retrieved papers (through searching of 3 databases and hand searching) by using the find duplicate function of the EndNote software program (Clarivate). Next, we selected papers by looking at the abstracts and titles and then checked for full-text paper eligibility. Full-text papers were evaluated for the final systematic review based on predefined inclusion and exclusion criteria. The eligibility of all studies was assessed by 2 blinded independent raters (EJG and CSB), and discrepancies were resolved by consultation with a third author (JJL).

### Inclusion and Exclusion Criteria

Studies were included if they satisfied the following criteria: (1) any type of studies that investigated the potential role of LLMs in the field of gastrointestinal endoscopy or gastroenterology, (2) studies published in English, and (3) studies in full-text format. The exclusion criteria were as follows: (1) studies that did not report the potential role of LLMs in the field of gastrointestinal endoscopy or gastroenterology, (2) case reports and review papers, (3) ineligible research objects (eg, animals or basic research), and (4) insufficient data regarding the potential role of LLMs. Only publications conducted on human participants were searched, and the bibliographies of relevant papers were also reviewed to identify additional studies. The search strategy to find the relevant papers is described in Textbox 1.

**Search strategy to find the relevant papers.**

**Database: MEDLINE (through PubMed; April 12, 2024)**

- #1 “large language model”[tiab] OR “LLM”[tiab] OR “foundation model”[tiab] OR “language vision model”[tiab] OR “GPT”[tiab] OR “ChatGPT”[tiab] OR “BERT”[tiab] OR “Claude”[tiab] OR “transformer”[tiab] OR “generative AI”[tiab]: 18148
- #2 “gastroenterology”[tiab] OR “gastrointestinal”[tiab] OR “endoscopy”[tiab] OR “gastroscopy”[tiab] OR “colonoscopy”[tiab]: 425845
- #3 #1 AND #2: 178
- #4 #3 AND English[Lang]: 132

**Database: Embase**

- #1 “large language model”:ab,ti,kw OR “LLM”:ab,ti,kw OR “foundation model deep”:ab,ti,kw OR “language vision model”:ab,ti,kw OR “GPT”:ab,ti,kw OR “ChatGPT”:ab,ti,kw OR “BERT”:ab,ti,kw OR “Claude”:ab,ti,kw OR “transformer”:ab,ti,kw OR “generative AI”: 20956
- #2 “gastoenterology”:ab,ti,kw OR “gastrointestinal”:ab,ti,kw OR “endoscopy”:ab,ti,kw OR “gastroscopy”:ab,ti,kw OR “colonoscopy”:ab,ti,kw: 624510
- #3 #1 AND #2: 242
- #4 #3 AND ([article]/lim OR [article in press]/lim OR [review]/lim) AND [English]/lim: 118

**Database: Cochrane Library**

- #1 (large language model):ab,ti,kw OR LLM:ab,ti,kw OR (foundation model):ab,ti,kw OR (language vision model):ab,ti,kw OR GPT:ab,ti,kw OR ChatGPT:ab,ti,kw OR BERT:ab,ti,kw OR Claude:ab,ti,kw OR transformer:ab,ti,kw OR (generative AI): 2550
- #2 gastoenterology:ab,ti,kw OR gastrointestinal:ab,ti,kw OR endoscopy:ab,ti,kw OR gastroscopy:ab,ti,kw OR colonoscopy:ab,ti,kw: 72568
- #3 #1 and #2: 123

### Data Extraction

Two evaluators (EJG and CSB) independently extracted the outcomes of all the finally included studies using Microsoft Excel sheet form (knowledge-based response evaluation, document summary or AI-generated draft response, overcome language barriers, identifying research questions, and combining multiple tasks including causal inference) and disagreements between the 2 evaluators were resolved by discussion or consultation with a third author (JJL).

### Methodological Quality Assessment

Risk of Bias in Non-Randomized Studies—of Interventions (ROBINS-I) was used to evaluate the quality of the identified studies [8]. Seven domains make up the ROBINS-I tool: “bias due to confounding,” “bias in selection of participants into the study,” “bias in classification of intervention,” “bias due to deviations from intended interventions,” “bias due to missing data,” “bias in measurement outcomes,” and “bias in selection of the reported result.” It is established that there is a “low,” “moderate,” “serious,” or “critical risk of bias” in each domain. The evaluation of each domain level determines the overall risk of bias judgment; a low risk suggests that the study is equivalent to a well-conducted randomized trial for all domains under consideration. A nonrandomized study with a “moderate risk of bias” has solid evidence, but it cannot be compared to a randomized trial. When there is a “serious risk of bias” in 1 or more domains but not a “critical risk of bias” in any 1 domain, it suggests that there are “serious risk of bias.” “Critical risk of bias” in at least 1 domain signifies that there is a significant danger of bias in the study, making it difficult to draw any meaningful conclusions [30]. Two evaluators (EJG and CSB) independently performed the methodological quality assessment, and disagreements between the 2 evaluators were resolved by discussion or consultation with a third author (JJL).

### Data Synthesis

Narrative synthesis was done because of heterogeneity in the specified aims and methodology in each included study.

## Results

### Potential Role of LLMs in Gastrointestinal Endoscopy

LLMs can transform gastrointestinal endoscopy by improving diagnostic accuracy, streamlining documentation, and enriching education and patient engagement [1]. By analyzing endoscopic imagery with precision and automating report generation, LLMs offer a layer of analysis that could reduce diagnostic errors and administrative burdens. Medical information retrieval is another potential role of LLMs [1]. They can quickly access and interpret large volumes of medical data and provide accurate answers to complex medical queries. This is particularly useful for answering rare or obscure medical questions and staying updated on the latest medical research. Through the same process stated earlier, they can support the diagnosis process and treatment recommendation. This can be used for clinical reasoning using LLMs with real medical cases. Furthermore, their application in creating interactive training materials and personalizing patient education presents an exciting frontier for the field [31]. One important benefit that should not be overlooked is emotional support [32]. By providing emotional support, answering patient queries, and assisting with daily tasks, they can help improve the overall patient experience and satisfaction [32].

### Systematic Review

**Figure 1:**
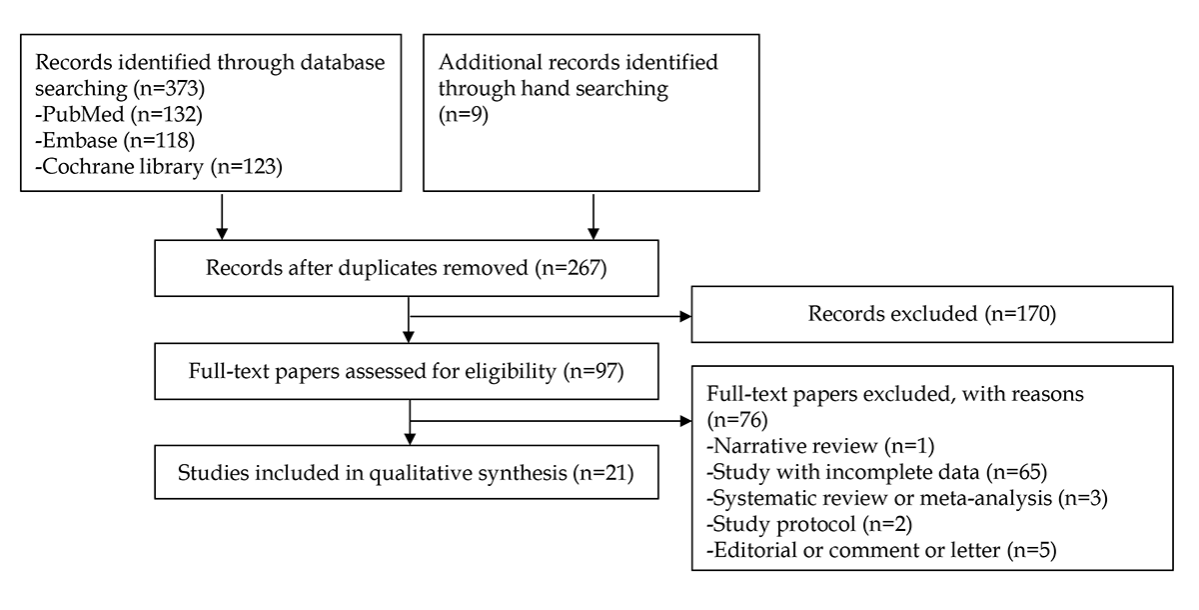
PRISMA (Preferred Reporting Items for Systematic Reviews and Meta-Analyses) flowchart of the study selection process.

A total of 373 studies were identified from the literature searching process on the 3 databases. Nine studies were additionally identified by manual screening of references. After excluding duplicate studies, additional papers were excluded after reviewing their titles and abstracts. Full-text versions of the remaining 97 studies were obtained and thoroughly reviewed based on the aforementioned inclusion and exclusion criteria. Among these, 76 papers were excluded because these papers did not meet the inclusion criteria (narrative review: n=1, study with incomplete data: n=65, systematic review or meta-analysis: n=3, study protocol: n=2, and editorial or comment or letter: n=5). Finally, 21 studies [9-29] for the potential role of LLMs in gastrointestinal disorders were included in the systematic review. A flowchart of the study selection process is shown in Figure 1.

Table 1 shows the summary of this systematic review. Each retrieved study [9-29] can be categorized by several topics, including knowledge-based response evaluation [9-22], document summary or AI-generated draft responses [23,24], overcome language barrier [25,26], identifying research questions [27], or combining multiple tasks including causal inference [28,29].

**Table 1:** Clinical summary of the included studies.

Outcome and study (year)			Nationality (institution)		Type of AI^a^ model		Study topic			Evaluation		Rating
**Knowledge-based response evaluation**												
	Lim et al (2024) [9]	Singapore		GPT-4		To evaluate whether the contextualized GPT model (using guidelines) could provide correct advice for screening and surveillance intervals for colonoscopy (62 example case scenarios)			3 gastroenterology fellows under the supervision of 2 senior gastroenterologists		The contextualized GPT-4 model outperformed the standard GPT-4 in all domains. No high-risk features were missed, and only 2 cases had hallucinations of additional high-risk features. A correct interval to colonoscopy was provided in the majority of cases. Guidelines were appropriately cited in almost all cases.	
	Munir et al (2024) [10]	United States		ChatGPT in June 2023		To assess the quality and perceived utility of chat-based AI responses related to 3 common gastrointestinal surgical procedures (cholecystectomy, pancreaticoduodenectomy, and colectomy)			45 surgeons		Overall, the most commonly assigned quality grade was “fair” or “good” for most responses (622/1080, 57.6%). Most of the 1080 total utility grades were “fair” (n=279, 25.8%) or “good” (n=344, 31.9%), whereas only 129 utility grades (11.9%) were “poor.” Overall, only 20% of the experts deemed ChatGPT to be an accurate source of information, whereas 15.6% of the experts found it unreliable. Moreover, 1 in 3 surgeons deemed ChatGPT responses as not likely to reduce patient-physician correspondence (31.1%) or not comparable to in-person surgeon responses (35.6%).	
	Rammohan et al (2024) [11]	United States		ChatGPT-4.0 and Google Bard (2023)		To assess the reliability and accuracy of LLMs^b^ in answering gastroenterology-related queries			2 independent reviewers		ChatGPT-4.0 demonstrated higher reliability and accuracy in its responses than Google Bard, as indicated by higher mean ratings and statistically significant *P* values in hypothesis testing. However, limitations in the data structure, such as the inability to conduct detailed correlation analysis, were noted.	
	Tariq et al (2024) [12]	United States		ChatGPT-3.5, ChatGPT-4, and Bard (version July 2023, queries ran on July 17, 2023)		47 common patient inquiries related to colonoscopy			2 reviewer gastroenterologists		In total, 43 of 47 (91.4%) responses graded as completely correct, whereas 4 of 47 (8.6%) responses by ChatGPT-4 were graded as correct but incomplete.	
	Gravina et al (2024) [13]	Italy		ChatGPT-3.5 and Perplexity AI		Performance in responding to questions from the 2023 Italian national residency admission examination (SSM23^c^) and comparing results and chatbots’ concordance with previous years’ SSMs			N/A^d^		In SSM23, ChatGPT-3.5 outperforms Perplexity AI with 94.11% of correct responses, demonstrating consistency across years. Concordance weakened in 2023 (κ=0.203; *P*=.148), but ChatGPT consistently maintains a high standard compared to Perplexity AI.	
	Atarere et al (2024) [14]	United States		ChatGPT, BingChat, and YouChat (April 2023)		15 questions on important colorectal cancer screening concepts and 5 common questions asked by patients			2 board-certified internal medicine physicians		ChatGPT and YouChat provided reliably appropriate responses to all 15 (100%) questions, while BingChat provided reliably appropriate responses to 13 of 15 (86.7%) questions and unreliable responses to 2 of 15 (13.3%) questions.	
	Gorelik et al (2024) [15]	Israel		Customized GPT		A custom GPT was developed to provide guideline-based management advice for pancreatic cysts			2 gastroenterologists (pancreatobiliary specialists) and a hepatobiliary surgeon		The custom GPT aligned with expert recommendations in 87% of scenarios. Initial expert recommendations were correct in 97% and 87% of cases, respectively. No significant difference was observed between the accuracy of custom GPT and the experts.	
	Cankurtaran et al (2023) [16]	Turkey		ChatGPT-4 (March 2023)		20 specific questions regarding IBD^e^			2 experts		Reliability and usefulness score as follows: mean 5.00 (SD 1.21) and mean 5.15 (SD 1.08), respectively (7-point Likert scale).	
	Gorelik et al (2023) [17]	Israel		ChatGPT (GPT-4)		Compliance with guidelines and accuracy about 20 clinical scenarios relevant to postcolonoscopy patient management			2 senior gastroenterologists		ChatGPT exhibited 90% compliance with guidelines and 85% accuracy, with a very good interrater agreement (Fleiss κ coefficient of 0.84; *P*<.01).	
	Ali et al (2023) [18]	United States		ChatGPT (launched in November 2022)		113 questions related to EGD^f^, colonoscopy, EUS^g^, and ERCP^h^			At least 2 board-certified or eligible advanced endoscopists		Moderate precision in answering questions about EGD (57.9% comprehensive), colonoscopy (47.6% comprehensive), EUS (48.1% comprehensive), and ERCP (44.4% comprehensive). Medical accuracy was highest for EGD (52.6% fully accurate) and lowest for EUS (40.7% fully accurate).	
	Lahat et al (2023) [19]	Israel		ChatGPT (November 2022)		To evaluate the performance of ChatGPT in answering patients’ 110 real-life questions regarding gastrointestinal health			3 experienced gastroenterologists		About treatments, the average (SD) accuracy, clarity, and efficacy scores (1 to 5) were 3.9 (0.8), 3.9 (0.9), and 3.3 (0.9), respectively. For symptom questions, the average (SD) accuracy, clarity, and efficacy scores were 3.4 (0.8), 3.7 (0.7), and 3.2 (0.7), respectively. For diagnostic test questions, the average (SD) accuracy, clarity, and efficacy scores were 3.7 (1.7), 3.7 (1.8), and 3.5 (1.7), respectively.	
	Lee et al (2023) [20]	United States		ChatGPT (January 30, 2023, version)		To evaluate the answers about 8 common questions about colonoscopy (compared to publicly available web pages of 3 randomly selected hospitals from the top 20 list of the US News & World Report’s Best Hospitals for Gastroenterology and Gastrointestinal Surgery)			4 gastroenterologists (2 senior gastroenterologists and 2 fellows)		Gastroenterologists rated ChatGPT answers similarly to non-AI answers in ease of understanding (AI: 5.0-6.4 vs non-AI: 4.8-5.8), with the AI mean scores higher than non-AI scores. Scientific adequacy scores were also similar (AI: 5.4-6.5 vs non-AI: 5.1-6.3; nonsignificant), with the AI mean score higher than non-AI 63% of the time. AI and non-AI answers received similar ratings regarding satisfaction with the answers (AI: 4.9-6.3 vs non-AI: 4.8-5.8; nonsignificant).	
	Samaan et al (2023) [21]	United States		March 14, 2023, version of GPT-4		To examine the accuracy and reproducibility of responses by GPT-4 to 88 patient nutrition questions related to IBD			2 IBD-focused registered dieticians		The model provided correct responses to 73 of 88 (83%) questions, with 61 (69%) graded as comprehensive. A total of 15 of 88 (17%) responses were graded as mixed with correct and incorrect or outdated data.	
	Henson et al (2023) [22]	United States		ChatGPT (version March 14, 2023)		Ability to respond appropriately to questions regarding gastroesophageal reflux disease (23 question prompts)			3 gastroenterologists and 8 patients		Appropriate responses (91.3%), although with some inappropriateness (8.7%) and inconsistency. Most responses (78.3%) contained at least some specific guidance. Patients considered this a useful tool (100%).	
**Document summary or AI-generated draft response**												
	Garcia et al (2024) [23]	United States		GPT-3.5 Turbo and GPT-4 (July to August 2023)		AI-generated draft response utilization rate across clinicians			162 clinicians		The mean AI-generated draft response utilization rate across clinicians was 20%. There were statistically significant reductions in the 4-item physician task load score derivative and work exhaustion scores.	
	Syed et al (2022) [24]	United States		Hybrid artificial neural network to concatenate and fine-tune BERT^i^ and Flair embeddings		To extract comprehensive clinical concepts from the consolidated colonoscopy documents			Validated using 300 colonoscopy procedures (the chart review was done by 4 reviewers [1 medical student and 3 trained data warehouse analysts] under the guidance of domain expert)		*F*_1_-scores of 91.76%, 92.25%, and 88.55% for colonoscopy, pathology, and radiology reports, respectively (5-fold cross-validation).	
**Overcome language barriers**												
	Yeo et al (2023) [25]	United States		ChatGPT and GPT-4				Evaluates ChatGPT and GPT-4’s ability to comprehend and respond to cirrhosis-related questions in English, Korean, Mandarin, and Spanish, addressing language barriers that may impact patient care	Native-speaking hepatologists		GPT-4 showed a marked improvement in the proportion of comprehensive and correct answers compared to ChatGPT across all 4 languages (*P*<.05). GPT-4 demonstrated enhanced accuracy and avoided erroneous responses evident in ChatGPT’s output.	
	Samaan et al (2023) [26]	United States		ChatGPT (January 30, 2023, version)				ChatGPT’s accuracy in responding to cirrhosis-related questions in Arabic and compared its performance to English (91 questions in Arabic and English were graded. Accuracy of responses was assessed using the scale.)	A transplant hepatologist fluent in both languages		The model provided 22 (24.2%) comprehensive, 44 (48.4%) correct but inadequate, 13 (14.3%) mixed with correct and incorrect or outdated data, and 12 (13.2%) completely incorrect Arabic responses. When comparing the accuracy of Arabic and English responses, 9 (9.9%) of the Arabic responses were graded as more accurate, 52 (57.1%) similar in accuracy, and 30 (33%) as less accurate compared to English.	
**Identifying research questions**												
	Lahat et al (2023) [27]	Israel		ChatGPT (December 15, 2023)				To evaluate the potential of ChatGPT for identifying research priorities in gastroenterology and provide a starting point for further investigation, we queried ChatGPT on 4 key topics in gastroenterology: IBD, microbiome, AI in gastrointestinal, and advanced endoscopy in gastroenterology	3 experienced gastroenterologists		On average, the questions were rated 3.6 (SD 1.4), with interrater reliability ranging from 0.80 to 0.98 (*P*<.001). The mean (SD) grades for relevance, clarity, specificity, and originality were 4.9 (0.1), 4.6 (0.4), 3.1 (0.2), and 1.5 (0.4), respectively (1-5 scale).	
**Combining multiple tasks including causal inference**												
	Zhou et al (2023) [28]	China		ChatGPT and GPT-4				To explore ChatGPT’s potential in disseminating gastric cancer knowledge, providing consultation recommendations, and interpreting endoscopy reports	Pre-established ground truth		GPT-4 model of ChatGPT achieved an appropriateness of 91.3% and a consistency of 95.7% in a gastric cancer knowledge test.	
	Wang et al (2022) [29]	United States		BioBERT		Causal inference of idiosyncratic DILI^j^ based on LiverTox			Domain experts		Accuracy of 0.92 and an *F*_1_-score of 0.84 for the DILI prediction. High concordance of 0.91 between the severity scores generated by model and domain experts.	

^a^AI: artificial intelligence.

^b^LLM: large language model.

^c^SSM: Scuole Specializzazione Medicina.

^d^N/A: not applicable.

^e^IBD: inflammatory bowel disease.

^f^EGD: esophagogastroduodenoscopy.

^g^EUS: endoscopic ultrasound.

^h^ERCP: endoscopic retrograde cholangiopancreatography.

^i^BERT: Bidirectional Encoder Representations from Transformers.

^j^DILI: drug-induced liver injury.

Most retrieved studies [9-22] have measured reliability by asking LLMs about their common or specified medical knowledge, such as common gastrointestinal disorders or gastrointestinal procedures (gastroesophageal reflux disease management, nutrition questions related to inflammatory bowel disease, screening and surveillance intervals for colonoscopy, guideline-based management advice for pancreatic cysts, or board examination tests). Evaluation of the performance in LLM was rated by expert endoscopists or gastroenterologists, and most of the studies have shown real-world applicability.

Another topic was the document summary or AI-generated draft responses [23,24], and these studies showed the potential for usability and improvement in assessments of the burden and burnout of medical specialists. Although the LLMs have the potential to serve as an adjunct source of information for patients, language barriers could impact the quality of response, and 2 studies [25,26] have pointed out this issue and the need for applications in diverse linguistic contexts for LLMs.

In the context of medical research, LLMs have been used in medical research to streamline literature reviews, enhance drug discovery processes, and assist in the design and analysis of clinical trials. Additionally, they support personalized medicine, biomedical data mining, and the interpretation of complex clinical information for improved decision-making and patient care. In this systematic review, one study was identified for this issue, and Lahat et al [27] tried to evaluate the potential of ChatGPT for identifying research priorities in gastroenterology and provide a starting point for further investigation. They showed that LLMs may be a useful tool for identifying research priorities in the field of gastroenterology, although more work is needed to improve the novelty of the generated research questions.

Since LLMs are capable of performing a wide range of tasks, studies have been conducted to align these functions in order to create a streamline and assess their performance. This necessitates a more complicated or occasionally customized LLM models. Wang et al [29] tried to establish a causal inference model of idiosyncratic drug-induced liver injury (DILI) based on the LiverTox database. BioBERT (fine-tuned model with biomedical-specific corpora, including PubMed abstracts and PubMed Central full-text papers) was used as a backbone model. To make BioBERT more specific for the DILI application, they further fine-tuned the BioBERT model with the extracted sentences from LiverTox [27]. Since the presumptive diagnosis of exclusion is the main diagnosis of DILI, it is time-consuming; however, this model has the potential to help differentiate it.

### Methodological Quality

The primary limitation was the question-and-answer interaction of LLMs to evaluate the measurement of outcomes in the “knowledge-based response evaluation” [9-22] or “overcome language barriers” studies [25,26]. This may differ from actual patient-physician conversations or practice situations and is subject to evaluator bias. Therefore, all the studies were rated in a moderate risk of bias in the “bias in measurement of outcomes” domain. Otherwise, all the other remaining domains were rated as “low risk of bias”: in the ROBINS-I tool methodology, evaluation was in the “knowledge-based response evaluation” studies (Table 2).

**Table 2:** Risk of bias evaluation (ROBINS-I^a^ assessment tool).

Study	Bias preintervention and at intervention domains				Risk of bias postintervention domains					Overall assessment of bias
	Bias due to confounding	Bias in selection of participants into the study	Bias in classification of intervention	Bias due to deviations from intended intervention		Bias due to missing data	Bias in measurement of outcomes	Bias in selection of the reported result		
Lim et al (2024) [9]	Low	Low	Low	Low		Low	Moderate	Low	Moderate	
Munir et al (2024) [10]	Low	Low	Low	Low		Low	Moderate	Low	Moderate	
Rammohan et al (2024) [11]	Low	Low	Low	Low		Low	Moderate	Low	Moderate	
Tariq et al (2024) [12]	Low	Low	Low	Low		Low	Moderate	Low	Moderate	
Gravina et al (2024) [13]	Low	Low	Low	Low		Low	Moderate	Low	Moderate	
Atarere et al (2024) [14]	Low	Low	Low	Low		Low	Moderate	Low	Moderate	
Gorelik et al (2024) [15]	Low	Low	Low	Low		Low	Moderate	Low	Moderate	
Cankurtaran et al (2023) [16]	Low	Low	Low	Low		Low	Moderate	Low	Moderate	
Gorelik et al (2023) [17]	Low	Low	Low	Low		Low	Moderate	Low	Moderate	
Ali et al (2023) [18]	Low	Low	Low	Low		Low	Moderate	Low	Moderate	
Lahat et al (2023) [19]	Low	Low	Low	Low		Low	Moderate	Low	Moderate	
Lee et al (2023) [20]	Low	Low	Low	Low		Low	Moderate	Low	Moderate	
Samaan et al (2023) [21]	Low	Low	Low	Low		Low	Moderate	Low	Moderate	
Henson et al (2023) [22]	Low	Low	Low	Low		Low	Moderate	Low	Moderate	
Garcia et al (2024) [23]	Low	Low	Low	Low		Low	Low	Low	Low	
Syed et al (2022) [24]	Low	Low	Low	Low		Low	Low	Low	Low	
Yeo et al (2023) [25]	Low	Low	Low	Low		Low	Moderate	Low	Moderate	
Samaan et al (2023) [26]	Low	Low	Low	Low		Low	Moderate	Low	Moderate	
Lahat et al (2023) [27]	Low	Low	Low	Low		Low	Low	Low	Low	
Zhou et al (2023) [28]	Low	Low	Low	Low		Low	Low	Low	Low	
Wang et al (2022) [29]	Low	Low	Low	Low		Low	Low	Low	Low	

^a^ROBINS-I: Risk of Bias in Non-Randomized Studies—of Interventions.

In terms of the studies with “document summary or AI-generated draft response” [23,24], “identifying research questions” [27], or “combining multiple tasks including causal inference” [28,29], the risk of “bias in measurement of outcomes” was minimal because pre-established ground truth exists or LLM’s answer was not the primary outcome. Therefore, all the studies were rated in a low risk of bias in these domains (Table 2).

## Discussion

### Principal Findings

This study explored the emerging role of LLMs in gastroenterology, especially for gastrointestinal endoscopy, providing a summary of recently published relevant papers through the systematic review process. In total, 21 studies from systematic review revealed the potential of LLMs for disseminating general medical knowledge, providing consultation recommendations, automatic generation of procedure reports, or causal inference of presumptive diagnosis of complex medical disorders.

### Benefits and Limitations

The integration of LLMs promises improved diagnostic accuracy, efficiency, enhanced education, and better patient engagement. In addition, LLMs can be used as a source of knowledge for medical staff and as a source of medical knowledge for patients [33]. It can be used as a questionnaire system to help medical staff and patients communicate, and it can also be used to generate papers and organize data for research purposes. This can be also used for quality improvement in medical practice. For example, LLMs can analyze electronic health records and better identify specified patients for alerts [34]. It is already proven to be good at arithmetic reasoning [35] and can also be used for the analysis of quality metrics, such as adenoma detection rate or polyp detection rate through the analysis of pathology reports of the patients. However, challenges such as ensuring data privacy, overcoming biases in training data, and maintaining human oversight highlight the need for careful implementation [1]. Additionally, integrating these technologies into clinical practice requires overcoming technical and cultural hurdles. A study that was recently published revealed that the LLM was found to amplify negative societal biases; overrepresent stereotypes, including problematic representations of minority groups; and exaggerate known disease prevalence differences between groups [36]. In their study, the authors noted that explicitly instructing the model to avoid bias or perform equitably is unlikely to produce the desired result and may even cause the model to overcorrect, resulting in an even worse bias [36,37]. Rather than relying solely on recommendations produced by the model, it appears essential that the model be connected to an independent, verifiable source of bias-free knowledge via retrieval-augmented generation [36,37]. Another major consideration is the likelihood of hallucinations. LLM is the general-purpose model. Prompt engineering, in which explicit instructions meant to exploit the optimal capabilities of LLMs are incorporated in addition to the question within the LLM input, can significantly improve LLM performance for specific tasks [38]. However, hallucinations may be common if we simply ask the question without any specific instructions. Fine-tuning or retrieval-augmented generation improves the goal-directedness of LLM [1]. Another limitation is the lack of clinical validation in the retrieved studies. Most selected studies are single-center pilot application formats with no large-scale studies or multicenter performance validation. Given that the performance of LLMs is constantly improving, this is likely to happen in the near future when they are ready for real-world clinical use.

### Future Directions

LLMs have the potential to help both patients and doctors in the future, such as communicating professionally with patients in clinical settings or participating in the process of summarizing and reasoning about various multimodal information during clinical diagnosis. In addition, it is likely to be applied to medical research, such as hypothesizing, testing, and planning experiments, and finally, in education, it can play a role in training medical staff by acting as a fictitious patient or taking questions on various medical situations. It is necessary to prepare for wise use in the future by overcoming the limitations of misinformation, hallucination, and ethical issues.

In terms of the technical aspects, although, the transformer-based vision model (language vision model) needs much more data compared to CNN, this does not have inductive bias, which is the limitation of CNN [39]. There is a potential for LLM or language vision model to cover all the current performance of CNN’s vision task in gastrointestinal endoscopy. These days, even LLMs with a focus on medicine are available [40,41]. Considering that medical practice is basically a multimodal task including history taking, visual diagnosis, data interpretation, and diagnosis reasoning, LLM-based foundation models that have multimodal function would be the next generation mainstream of AI model in medical practice [1]. LLMs, as a form of generative models, along with other generative models like generative adversarial networks, diffusion models, and variational autoencoders, are evolving to include creative features that are being integrated into the LLM framework. It is important to acknowledge that while LLMs are making strides in medical applications, issues such as model hallucination and unexpected biases remain challenges that require vigilant attention and ongoing research to mitigate. As we continue to witness the introduction of new LLM models with larger parameters and optimized performance, it is clear that these technologies are becoming indispensable tools in real-world clinical settings. Their potential to enhance medical practice is immense; yet, it is crucial to approach their integration with care to ensure patient safety and uphold ethical standards. The future directions of LLMs in gastroenterology, powered by advancements in processing units and innovative platforms, hold the promise of transforming the landscape of medical diagnostics and treatment, making health care more effective, personalized, and accessible to patients around the globe.

### Limitation of This Systematic Review

Due to the different objectives and primary outcomes of each retrieved study, a meta-analysis (quantitative synthesis) was not feasible, which is a drawback of this systematic review process. Consequently, it was impossible to evaluate publication bias. A systematic review, however, showed that the main outcomes of each study divided by several categories, which LLMs are currently in use for the clinical research, which primary tasks are assessed, and what the limitations of each study are. However, in terms of future research perspectives, the outcomes of this study can assist in comprehending the current state of gastroenterology research using LLMs and designing a study that complements the existing limitations.

### Conclusions

The potential of LLMs to revolutionize gastrointestinal endoscopy is immense, offering improvements in diagnostic accuracy, operational efficiency, and patient care. The successful integration of LLMs hinges on addressing data privacy concerns, ensuring quality data, and fostering interdisciplinary collaboration. As we embrace these technological advancements, the future of endoscopy looks toward a more informed, efficient, and patient-centered approach.

## Appendix

Multimedia Appendix 1 PRISMA (Preferred Reporting Items for a Systematic Review and Meta-Analyses) 2020 checklist.

## Abbreviations

AI: artificial intelligence
BERT: Bidirectional Encoder Representations from Transformers
CNN: convolutional neural network
DILI: drug-induced liver injury
LLM: large language model
PICO: population, intervention, comparator, and outcome
PRISMA: Preferred Reporting Items for a Systematic Review and Meta-Analyses
ROBINS-I: Risk of Bias in Non-Randomized Studies—of Interventions

## Data Availability

All data generated or analyzed during this study are included in this published paper.
